# 

**Published:** 1891-01-31

**Authors:** 


### NOTICE TO CORRESPONDENTS.

All MS., Letters, Books for Review, and other matters intended for the Editor should be addressed The Editor, The Lodge, Porchester Square, London, W.

The Editor cannot undertake to return rejected M.S., even when accompanied by stamped directed envelope.

**Notice to Advertisers and Subscribers.**

All Advertisements, Orders for Copies of The Hospital, and Business Communications should be addressed to The Publisher, *not to the Editor*, The Hospital, 140, Strand, W.C.

The Cost of Subscription, post free, is as follows:—Per week, 1½d.; per quarter, 1s. 8d.; per half-year, 3s. 3d.; per annum, 6s. 6d. Foreign:—Per annum, 8s. 8d.; payable, in all cases, in advance.

**Bound Volumes of "The Hospital."**

Vol. VI., containing full page portraits of T.R.H. the Prince and Princess of Wales, and Miss Florence Nightingale, is still obtainable. Vol. VII. and all the earlier volumes are also for sale.

Orders will be received for Vol. VIII., 4s. post free. Cases for binding may be had of the Publisher, at 1s. 6d. each.

**To Residents, Secretaries, and Matrons.**

The Editor will be glad to have the Regulations and each Report as issued by Asylums, Hospitals, Convalescent and Nursing Institutions, as well as those of other Charities, and an early copy in duplicate of all Appeals and Festival Notices, etc., addressed to *The Editor, The Lodge, Porchester Square, London, W.* Any superintendent, secretary, matron, or other chief official who regularly sends such information may be registered, on application to the Publisher, to receive free of cost a cover for binding The Hospital in half-yearly volumes.

### BURDETT'S HOSPITAL ANNUAL

May be obtained of the Publisher, The Hospital Offices, 140, Strand, W.C., price 2s. 6d. limp boards, or 3s. 6d. cloth. All orders must be accompanied by a remittance. (Note: The edition for 1891 will not be published until April, the current number containing latest information procurable to that date).

### Scraps and Gleanings.

Mr. F. J. Horniman has contributed fifty guineas to the Royal Free Hospital, Gray's Inn Road.

The Queen has presented a supply of cast linen to a number of the principal hospitals of the metropolis.

The Skinners' Company have given £50 towards the funds of the National Physical Recreation Society.

Lord Greville, who has had the misfortune to be bitten by a dog, has placed himself under the care of M. Pasteur.

The Surrey County Council have appointed Dr. Seaton as medical officer of health for the county, at a salary of £800 a-year.

The death is announced of Dr. Edward John Waring, a physician who devoted his life to the study of the medicinal resources of India.

The Queen has sent twenty pheasants to the National Hospital for the Paralysed and Epileptic. Lord Rothschild has sent a similar gift.

A Berlin telegram states that an English bacteriologist, Mr. E. H. Hankin, who is now studying Dr. Koch's cure, has discovered a cure for anthrax.

A cot, to be known as the "Henry White Bed," is to be endowed in the children's ward at King's College Hospital, in memory of the late Rev. Henry White.

Viscount Grimston, M.P., has been elected chairman of the National Hospital for Diseases of the Heart and Paralysis in the place of the late Earl of Glasgow.

A bazaar in aid of St. Mary's Seaside Home of Rest for the Blind, St. Leonards-on-Sea, will be held in the first week in February at Oxford Hall, Bethnal Green.

A ball in aid of the funds of the Cumberland Infirmary has been given in the Town Hall, Carlisle. The attendance numbered over 300. The ball was in every way an immense success.

On the 13th inst. an entertainment was given to the patients in Brompton Hospital. Miss Wilson Theobald organised the programme, which included both vocal and instrumental music.

The Duke of Portland has consented to preside at the twelfth festival dinner of the East London Hospital for Children, Shadwell, which will be held at the Hotel Métropole, on Monday, March 2nd.

The Duke of Clarence and Avondale has consented to preside at the festival dinner at the Hotel Métropole, early in May, in aid of the funds of the Royal Hospital for Women and Children, Waterloo Bridge Road.

An amateur performance of "Les Cloches de Corneville," in aid of the funds of the Surbiton Cottage Hospital and Kingston Provident Dispensary, will be given at Surbiton early in February. The Duchess of Teck has signified her intention of being present.

His Grace the Duke of Fife, K.T., President of the Hospital for Sick Children, Great Ormond Street, Bloomsbury, will take the chair at the annual dinner to be held on Wednesday, 18th March, at the Hotel Métropole.

The sixth grand annual ball in aid of the funds of the Borough Hospital, was held in the Town Hall, Bootle. The decorations and arrangements were carried out in a manner to give general satisfaction, and in point of numbers the ball was one of the most successful ever held in the borough.

Mr. T. Bedford Bolitho, P.M., sent to be read at the West Cornwall Infirmary annual meeting his scheme for a Convalescent Home in memory of his late father. He proposes a first endowment of £10,000 for eight beds, and adds that more money is available to make the "Edward Bolitho Convalescent Home" thoroughly useful.

Two doctors of Nantes have been attempting to cure consumption by means of goats' blood. Acting on the well-known fact that the goat is not liable to the disease, they have transfused the blood of the animal into several hospital patients. Remarkable improvement is said to have taken place in the condition of patients thus treated.

An excellent amateur performance of Lecocq's comic opera "Pepita," was given at St. George's Hall last Saturday evening, in aid of the funds of the Samaritan Free Hospital for Women and Children, Marylebone Road. The hall was well filled, and the performance went with plenty of life and sparkle, and altogether the rendering and mounting of the opera reflected great credit upon those concerned.

A consumptive patient in the Bellevue Hospital, New York, is stated to have been cured by Dr. Koch's method. The first injection of the lymph was administered to him on December 24th, and the last injection took place on January 2nd. There was no reaction, and the patient showed continuously rapid improvement until the 14th inst., when he left the hospital apparently cured, and having gained 18 pounds in weight.

Dr. F. Norton Manering, the President of the Board of Health, has written a report on the condition of the leper patients at the Little Bay Hospital. He states that all the European patients are in a comparatively early stage of disease, three of them being strong men and the other a boy in good bodily health, though somewhat helpless from one hand being crippled. The patients were attended to, and their sores were dressed by a qualified hospital attendant who had had four years' experience.

A meeting, convened by the Lord Mayor, was held at the Mansion House on behalf of the Building Completion Fund of the Royal Free Hospital. The Lord Mayor, in opening the proceedings, said that three sides of the building had been re-erected on the most approved principles, but the front portion, which included the casualty-room and the officers' apartments, was too small as well as unsuitable. More room was urgently needed in the accident ward, as this was the hospital to which came all people injured at the terminal stations of the three largest railways in England. At the close of the meeting, subscriptions amounting to £2,000 were announced.

### BOOKS RECEIVED.

Bemrose and Son.

"Scenes in the Life of a Nurse." Sister Eva.

Cassell and Co.

"Hygiene and Public Health." B. Arthur Whitelegge, M.D. Price, 7s. 6d.

Dorman, M. J. (Philadelphia).

"I. The Prevention of the Short Leg of Hip Disease; II. The After Treatment of Hip Disease." A. B. Judson, M.D. (New York). Reprinted from the Transactions of the American Orthopædic Association, Sept., 1889.

Griffin and Co., Charles.

"Food and Dietaries: A Manual of Clinical Dietetics." R. W. Burnet, M.D.

Lewis, H. K.

"Pocket Medical Lexicon." John M. Keating, M.D., and Henry Hamilton.

Longmans, Green, and Co.

"Aphorisms in Applied Anatomy (or Anatomy for the Final Examination) and Operative Surgery." Thomas Cooke, B.A., B.Sc., &c. Price, 3s. 6d.

Philip and Son, George.

"Educational Annual for 1891." Price, 2s. 6d.

### THE STUDENT OF MEDICINE.

[All communications for this Supplement should be addressed to THE EDITOR of THE HOSPITAL, at 140, Strand, with the words, "Students' Column," written in the left hand top corner of the envelope.]

### NOTES FROM THE SCHOOLS.

### EDINBURGH UNIVERSITY.

The Union debate held on January 14th was not interesting from a medical point of view, the motion being that "The Churches of England and Scotland should be forthwith Disestablished." The discussion was fairly well kept up, though towards the end the number of members present was considerably thinned. The motion was lost by 13.

The official report of the University shows the numerical strength of the University for the past year—matriculated students 3,503, against 3,602 in 1889, 3,160 in 1880, and 1,768 in 1820. Of these, 1,979 are in the faculty of medicine. Of the medical students Scotland sends 814, England and Wales 687, British Colonies 270, India 99, Ireland 58, and foreign countries 51. Of the graduates 54 were M.D.'s, and 210 were M.B. C.M.'s.

The Union Ball was held on Tuesday, January 20th, in the Union. There were about three hundred present, the ball being even a greater success than last year. Much praise is due to the Amusement-Committee for the excellent arrangements.

The University members of the Edinburgh City Volunteer Artillery now constitute a Battery in that Corps, under the command of Lieut. Professor Cossar Ewart.

Sir Charles Russell has written to the University Liberal Association thanking his 805 supporters in the recent Rectorial election.

Members of the "Students' Representative Council" have been busy this week collecting the "shilling voluntary tax" from students, for the Council funds.

The Organ Recital given by Mr. John Grieg for the Professor of Music, was better attended than the last one, the Music Class Room being well filled. The whole programme was very well received. The second item, a Prelude and chorus, was loudly applauded, and the Romance given as the seventh piece was encored.

### APPOINTMENTS.

At the London Hospital Medical College, Henry Percy Dean, B.Sc.Lond., M.B., has been appointed surgical registrar; G. J. Schorstein, M.B., B.S.Oxon., has been appointed assistant demonstrator of anatomy. At the Denbighshire Infirmary, Denbigh, J. Lewis Owen, M.R.C.S., has been appointed house surgeon. At the Alnwick Infirmary, Benjamin Sweeten, M.B., C.M., has been appointed house surgeon. At the Staffordshire General Infirmary, George Tate, M.B., C.M.Edin., has been appointed assistant house surgeon. At the Paddington Infirmary, London, Victor Hugo Edward, M.B., B.S.Lond., has been appointed assistant medical superintendent. At the Royal Free Hospital, Wilmott Ewans, M.D., B.S., B.Sc.Lond, F.R.C.S., has been appointed registrar. At University College, London, C. Byron Turner, M.R.C.S., L.R.C.P., has been appointed demonstrator of anatomy. At the Norfolk and Norwich Hospital, Reginald E. Crosse, M.R.C.S., L.R.C.P. (Bart's.), has been appointed house surgeon. At the Suffolk General Hospital, C. S. Kilner, M.D., C.N.Edin., has been appointed assistant medical officer. At the Carmarthen Infirmary, Edward Somerset, M.R.C.S., L.R.C.P.Lond, L.S.A., has been appointed house surgeon. At the Workhouse at Edmonton, W. B. Benjafield, M.B.Edin., M.R.C., M.R.C.S., has been appointed medical officer. At the Workhouse of the Sturminster Union, J. C. Leach, M.D. Durh., M.R.C.S., has been appointed medical officer. At the Workhouse of the Cardiff Union, J. D. Williams, M.D.Edin., has been appointed assistant medical officer. At the Stafford Union, Edward Woodhouse, M.B., C.M.Edin., has been appointed medical officer.

### VACANCIES.

The following vacancies are announced this week, the amount of salary in each case being given after the name of the institution:—

**Resident Medical Officers.**

General Hospital, Birmingham, Assistant House Surgeon—Board, &c.

Vacancies—(*continued*).

Central London Ophthalmic Hospital, Gray's Inn Road, House Surgeon—Rooms, &c.
City Hospital for Infectious Diseases, Newcastle-on-Tyne, Resident Medical Assistant—Salary, £50 and board.
Royal Portsmouth, Portsea, and Gosport Hospital, House Surgeon—Salary, £70, board, &c.
Royal Orthopædic Hospital, Oxford Street, Resident House Surgeon and Apothecary—Salary, £100, with partial board.
Wirral Children's Hospital, Woodchurch Road, Birkenhead, Resident House Surgeon (lady or gentleman)—Salary, £50, and board.
City of London Lunatic Asylum, near Dartford, Kent, Assistant Medical Officer—Salary, £150, and board.
Dundee Royal Lunatic Asylum, Clinical Assistant—board.
Dundee Royal Lunatic Asylum, Assistant Medical Officer—Salary, £100, and board.
Lancashire County Asylum, Rainhill, near Liverpool, Pathological Assistant Medical Officer—Salary, £200, board, &c.

**Non-Resident Medical Officers.**

Charing Cross Hospital, Assistant Surgeon.
Chelsea Hospital for Women, Physician to the Out-patient Department.
Metropolitan Hospital, Kingsland Road, Dental Surgeon.
National Hospital for the Paralysed and Epileptic, Queen's Square, Bloomsbury, Surgeon.
Parish of Tongue, Sutherlandshire, Medical Officer—Salary, £165
St. George's Hospital, Lecturer on Midwifery.
University of Sidney, N.S.W., Demonstrator of Physiology—Salary, £350.
Winchomb Union, Winchomb, Medical Officer for the No. 2, or Vale District—Salary, £65, with additional fees.
Worcester General Infirmary, Physician.

### PASS LISTS.

**Royal College of Physicians and Surgeons.**

The following gentlemen passed the second examination of the Board in anatomy and physiology at a meeting of the examiners on the 16th inst.: Physiology only. B. E. Church and P. T. Jones, St. Bartholomew's Hospital; C. M. Welburn, A. W. C. Lindsay, C. W. Smith, and J. A. Edsell, St. Bartholomew's Hospital and Mr. Cooke's School of Anatomy and Physiology; H. Andreae, University College; W. F. C. Dowding and H. N. N. Dodd, St. George's Hospital and Mr. Cooke's School of Anatomy and Physiology; E. E. S. Silver. J. G. B. Colman, E. S. Tuck, and A. B. Oreak, Guy's Hospital; W. Pell, G. E. Kinnersley, F. W. Mason, and W. G. Macauley, St. Thomas's Hospital; G. R. Swinhoe, St. Thomas's Hospital and Mr. Cooke's School of Anatomy and Physiology; F. Whitelaw, St. Mary's Hospital; G. E. Williams, London Hospital; W. H. Gossage, Westminster Hospital; and C. M. S. Farnum, King's College.

### ATHLETICS.

**Football.**

Rugby Rules, Jan. 14th.

*East Sheen v. St. Thomas's Hospital.*—These teams, playing under Rugby Union Rules, should have met on Wednesday afternoon at the Athletic Grounds, Richmond. In accordance with instructions the Hospitalers' team duly put in an appearance at the rendezvous, but though the East Sheen team was advertised only four of them put in an appearance. As matters turned out it was a waste of time on both sides, for though the ground was clear of snow it was still so hard that play, more especially under the Rugby code, was completely out of the question, there being far too much "bone" in the ground, and consequently the match remains in abeyance.

January 24th.

*St. Mary's Hospital* was beaten by *Caledonian*, at Wormwood Scrubbs, by one try to one try one goal. *St. Thomas's Hospital* beat *Clapham Rovers*, at Wandsworth, by one goal one try to one try. *University College Hospital* beat *Surrey Wanderers*, at Park, by one goal one try to nil. *London Hospital* and *Upper Clapton*, drawn.

Hospitals Cup.—On Monday, the 26th, the annual competition for the Hospitals Cup began The matches are played on the Athletic Ground at Richmond. St. Thomas's (holders) beat St. Mary's by four goals and two tries to one try. The first goal was obtained from a penalty kick, two others were placed from tries, and one was dropped. On Tuesday, the 27th, Middlesex Hospital beat Bartholomew's by one goal to nil.

Association Rules, Jan. 14th.

Guy's Hospital were defeated by the Clapham Rovers on the latter's ground at Wandsworth, by two goals to one.

January 24th.

*St. Mary's Hospital* was beaten by *Clapton*, at Raynes Park, by three goals to four. *St. Bartholomew's Hospital* beat *Upton Ivanhoe*, at Upton, by two goals to one. *Middlesex v. St. George's*, played on Saturday at Ealing Dean, and resulted after a fast and evenly contested game in favour of the former by two goals (Wilkinson, Lewis) to one. In addition to those named, Huddlestone played well, as did the backs on both sides. Middlesex were one short throughout.

